# Duplicating Complete Dentures with Conventional and Digital Methods: Comparisons of Trueness and Efficiency

**DOI:** 10.3390/dj10030035

**Published:** 2022-03-01

**Authors:** Li Chen, Deli Li, Jianfeng Zhou, Wei-Shao Lin, Jianguo Tan

**Affiliations:** 1 Department of Prosthodontics, Peking University School and Hospital of Stomatology & National Clinical Research Center for Oral Diseases & National Engineering Research Center of Oral Biomaterials and Digital Medical Devices & Beijing Key Laboratory of Digital Stomatology, Beijing 100081, China; chenli@pkuss.bjmu.edu.cn (L.C.); zhoujianfeng@pkuss.bjmu.edu.cn (J.Z.); kqtanjg@bjmu.edu.cn (J.T.); 2 Second Clinical Division, Peking University School and Hospital of Stomatology & National Clinical Research Center for Oral Diseases & National Engineering Research Center of Oral Biomaterials and Digital Medical Devices & Beijing Key Laboratory of Digital Stomatology, Beijing 100101, China; leedelee@bjmu.edu.cn; 3 Department of Prosthodontics, Indiana University School of Dentistry, Indianapolis, IN 46202, USA

**Keywords:** complete denture, 3D printing, cone-beam CT, CAD-CAM

## Abstract

Background: A complete denture (CD) can be duplicated with a conventional or digital protocol. However, there are no comparative studies of these methods. This study aimed to compare the trueness and efficiency of conventional and digital CD duplication methods. Methods: A mandibular CD was digitized as the virtual reference model and duplicated using five methods (*n* = 10). The trueness (root mean square (RMS)) was calculated for the whole denture and across the dentition, cameo denture extension, and intaglio portions. The manual labor time spent during denture duplication was also recorded at different steps. The trueness and labor time comparisons were statistically analyzed among the five groups (α = 0.05). Results: The conventional group was the least true with the largest RMS (mean, 95% CI) in all of the comparisons. The four digital groups yielded similar trueness values across the dentition, cameo denture extension, and intaglio areas, yet they had a significant difference in the whole denture comparison between the Digital-CBCT-SLA printer (0.17, 0.15–0.19 mm) and Digital-Laboratory Scanner-SLA printer (0.13, 0.11–0.15 mm). The conventional protocol required longer trimming and finishing time (7.55 ± 1.02 min), as well as total labor time (27.64 ± 1.72 min) than the other four digital techniques. Conclusions: The conventional CD duplication method was less true and efficient than digital techniques.

## 1. Introductions

A clinically satisfactory complete denture (CD) can be duplicated for multiple indications. A duplicated CD can preserve the diagnostic information, such as tooth size and arrangement, occlusal schemes, denture extension, and morphology of denture-bearing areas [1,2]. Moreover, it can be used as an individualized tray to obtain the definitive impression, record the maxillomandibular relationship, and transfer the esthetic information to the dental laboratory technicians [3,4,5,6]. When radiopaque markers are incorporated, a duplicated CD can serve as a radiographic template to obtain digital diagnostic information for implant planning and be modified into a surgical template for computer-assisted surgery [7,8].

Computer-aided design and computer-aided manufacturing (CAD/CAM) technology is receiving popularity in prosthetic dentistry. In addition, it has reported advantages of user-friendliness, speed, elimination of manual labor, and production of high-quality prostheses. Clinicians can choose conventional or digital methods to duplicate a clinically satisfactory CD. The conventional protocol usually utilizes irreversible hydrocolloid or elastomeric impression materials to create a mold, which is the negative representative of the CD. In addition, the auto-polymerizing acrylic resin is poured into the mold to complete the duplication process. Stock trays or denture flasks are often used to contain and support impression materials [1,2,3,9,10]. The conventional method is labor-intensive and time-consuming [1]. When the irreversible hydrocolloid impression material is used, the entire duplication procedure should be completed rapidly to avoid distortion [11]. Moreover, a CD can be duplicated with digital methods. First, the CD is digitized and converted into a digital file format, which is compatible with computer-aided design (CAD) and computer-aided manufacturing (CAM) processes. Both cone-beam computed tomography (CBCT) and optical scanner can be used to digitize a CD [12]. When CBCT is used, the CD is digitized in the Data Imaging and Communications in Medicine (DICOM) file format, then converted into Standard Tessellation Language (STL) or Wavefront Object (OBJ) file formats [13,14,15]. With an optical scanner, the intaglio and cameo surfaces of a CD can be scanned separately, and then merged into a single STL file [16] or scanned continuously by rotation of the CD [17]. After digitization, a CD can be fabricated by subtractive (milling) or additive (3D printing) manufacturing technologies. Various 3D printing technologies have been used to fabricate removable dental prostheses, including digital light processing (DLP), stereolithographic (SLA), fused deposition modeling (FDM), and Polyjet [15,16,17,18,19,20,21].

Several studies have investigated the effects of manufacturing technologies on complete denture fabrication. For the denture base adaptation, the digitally manufactured complete dentures (3D-printed and milled) showed better adaptation than [21,22,23,24] or a similar fit with [20,25,26,27] conventionally manufactured ones. Consequently, a clinical study showed that the milled denture base exhibits higher retention than the conventional heat-polymerized denture base [28]. Furthermore, the digital denture protocol yielded significantly less clinical chairside time, and laboratory and overall costs [29]. In contrast, very few studies have focused on the effects of manufacturing methods on denture duplications.

The purpose of this study was to compare the trueness and efficiency of conventional and digital methods for complete denture duplications. The null hypotheses were that the trueness and efficiency would not be affected by denture duplication methods.

## 2. Material and Methods

A milled mandibular CD was selected as the master reference. The master reference CD was lightly coated with anti-glare spray (Helling 3D Anti-Glare Scan Spray; Laser Design, Minneapolis, MN, USA) with an average particle size of 2.8 μm, and then scanned with a laboratory scanner of accuracy level of 15 μm (7Series; Straumann, Andover, MA, USA). All of the scans were completed in a controlled environment under the humidity of 40% to 60% and temperature of 23 to 25.5 °C. The cameo surface was scanned first, and then the intaglio surface was scanned subsequently. Although the cameo and intaglio surfaces were scanned separately, the areas within 5–10 mm adjacent to the denture extensions were included in both scans. These overlapping areas were used to merge two separate scans in a 3D inspection software (Geomagic Control X; 3D systems, Rock Hill, SC, USA) to compose a digital master reference CD file in the STL format. Two lines were drawn on the merged file in the reverse engineering software (Geomagic Wrap X; 3D systems). One was used to separate the dentition and denture extension on the cameo surface, and the other was drawn between the cameo and intaglio surfaces. The digital master reference CD STL file was separated into dentition, cameo denture extension, and intaglio portions. Therefore, three additional digital master reference STL files were created for the subsequent trueness comparison at each respective area.

The master reference CD was duplicated using five different protocols (*n* = 10) (Table 1). The sample size in each group (*n* = 10) was based on an estimation of the effect sizes at 0.25, type I error at α = 0.05, and type II error at β = 0.80. Group 1 was the conventional duplication method. For each duplication, irreversible hydrocolloid impression material (Jeltrate regular set; Dentsply Sirona, York, PA, USA) was mixed with a spatula for 15 s with distilled water (following the manufacturer’s recommendation on powder and water ratio), and then for 40 s with a vacuum mixer (Vacuum Power Mixer Plus and Combination Unit; Whip Mix, Louisville, KY, USA). The mixed material was placed in the lower half of the duplication flask (Denture Duplicator–Flask; Lang Dental Manufacturing Company Inc., Wheeling, IL, USA). The master reference CD was placed in the irreversible hydrocolloid impression material with denture teeth perpendicular to the bottom of the flask. Following the trimming of the excessive impression material, separating fluid was applied on the denture and the surrounding impression material. The upper half of the duplication flask and intaglio side of the master reference CD was filled with the irreversible hydrocolloid impression material (Jeltrate regular set). The duplication flask was closed, and the closure screw was hand-tightened to create an impression mold. Clear acrylic resin (Caulk Orthodontic Resin; Dentsply Sirona, York, PA, USA) was mixed according to the manufacturer’s recommendation and poured into the impression mold. The duplication flask was closed and secured in a pneumatic polymerization unit (Acri-Dense Pneumatic Curing Unit; GC America, Alsip, IL, USA) under the 20 psi air pressure for 20 min. The duplicated CDs were trimmed and polished with the laboratory instruments (Ultra Denture Kit, Brasseler USA Dental, Savannah, GA, USA). The manual labor time spent during denture duplication was recorded at different steps.

**Figure 1:**
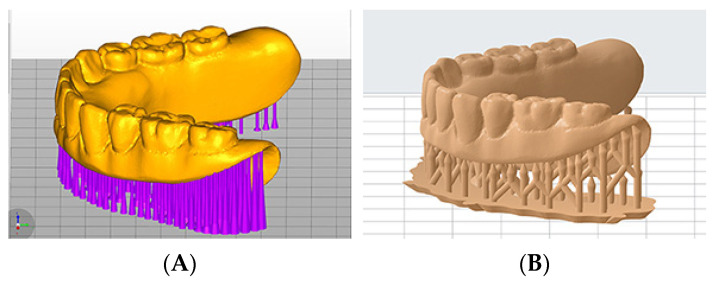
Printing settings for two types of printers. (**A**) Printing setting for DLP printer. (**B**) Printing setting for SLA printer.

Digital duplication methods were also used in this study. For Groups 2 and 3, the master reference CD was scanned with the CBCT imaging at fields of view (FOV) of 80 × 80 mm, 75 kV, and 2.0 mA (3D Accuitomo 170; J. Morita USA, Irvine, CA, USA). The volumetric dataset was saved in the data imaging and communications in medicine (DICOM) file format. The DICOM files were imported into an implant planning software (coDiagnostix; Straumann, Andover, MA, USA) and converted into STL files of digitized master reference CD (Figure 1A). The master reference CD was scanned 10 times with the aforementioned CBCT imaging protocol, and the resulting files were used to additively manufacture duplicated dentures for both Groups 2 and 3. In Group 2 (*n* = 10), the duplicated dentures were 3D-printed using a DLP 3D printer (MAX X43; Asiga, Alexandria, Australia) and light-polymerizing resin (VeriGuide OS Resin; Whip Mix, Louisville, KY, USA). In Group 3, the duplicated dentures were 3D-printed using a SLA 3D printer (Form 2; Formlabs Inc., Somerville, MA, USA) and light-polymerizing resin (Surgical Guide Resin; Formlabs Inc.).

**Figure 2:**
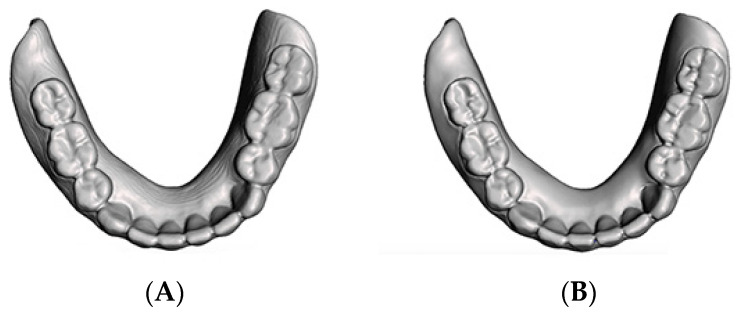
Occlusal view of the digital denture. (**A**) Digital denture produced by the CBCT scan and convert protocol. (**B**) Digital denture produced by the optical scan and merge technique.

For Groups 4 and 5, the master reference CD was scanned 10 times according to the previous laboratory scanner scanning protocol, and the resulting files were used to additively manufacture duplicated dentures (Figure 1B) for both Groups 4 and 5. In Group 4 (*n* = 10), the duplicated dentures were 3D-printed using a DLP 3D printer (MAX X43; Asiga, Alexandria, Australia) and light-polymerizing resin (VeriGuide OS Resin; Whip Mix, Louisville, KY, USA). In Group 5, the duplicated dentures were 3D-printed using a SLA 3D printer (Form 2; Formlabs Inc., Somerville, MA, USA) and light-polymerizing resin (Surgical Guide Resin; Formlabs Inc.). All of the digitally duplicated sample dentures were 3D-printed with an occlusal plane parallel to the printing plate (Figure 2A,B). In the DLP 3D-printer groups (Groups 2 and 4), 0.3 mm touchpoint sizes were used. In the SLA 3D-printer groups (Groups 3 and 5), 0.9 mm touchpoint sizes were used. The manual labor time spent during denture duplication was recorded at different steps.

All of the study sample dentures were scanned according to the previous laboratory scanner scanning and software program processing protocols to create digitized files in STL file format. First, the STL files of the digitized study sample dentures were aligned manually with the digital master reference CD STL file by selecting three corresponding matching points in a 3D scanning software (Geomagic Wrap 2016; 3D Systems, Rock Hill, SC, USA). Subsequently, each study sample denture file was separated into dentition, cameo denture extension, and intaglio portions, according to the previous software program processing protocol. To determine the trueness (root mean square (RMS), measured in mm) of conventional and digital methods for complete denture duplications, comparisons between the digitized study sample dentures and digital master reference CD STL files were conducted for the whole denture and across the dentition, cameo denture extension, and intaglio portions. All of the study sample STL files were superimposed to the corresponding reference STL files using voxel-based best-fit alignment in the surface matching software (Geomagic Control X; 3D Systems, Rock Hill, SC, USA). Color maps were also produced to demonstrate the qualitative three-dimensional differences between the test and reference files.

Normal distribution of the data and homogeneity of the variances were accessed with the Shapiro–Wilk normality test and Levene’s Test for Equality of Variances, respectively, prior to further statistical analysis. To analyze the differences in trueness (RMS) among the study groups, the multivariate analysis of variance (MANOVA) was used for the measurements at the whole denture and across the dentition, cameo denture extension, and intaglio portions. Following MANOVA, a one-way analysis of variance (ANOVA) was used to determine the effect of duplicating protocols on each dependent variable. Tukey’s post-hoc HSD test was used for multiple comparisons among the groups. The labor time spent for each group was also analyzed using the one-way ANOVA and Tukey’s post-hoc HSD test. A statistical software (SAS version 9.4; SAS Institute Inc., Cary, NC, USA) was used for all of the statistical analyses (α = 0.05).

## 3. Results

**Figure 3:**
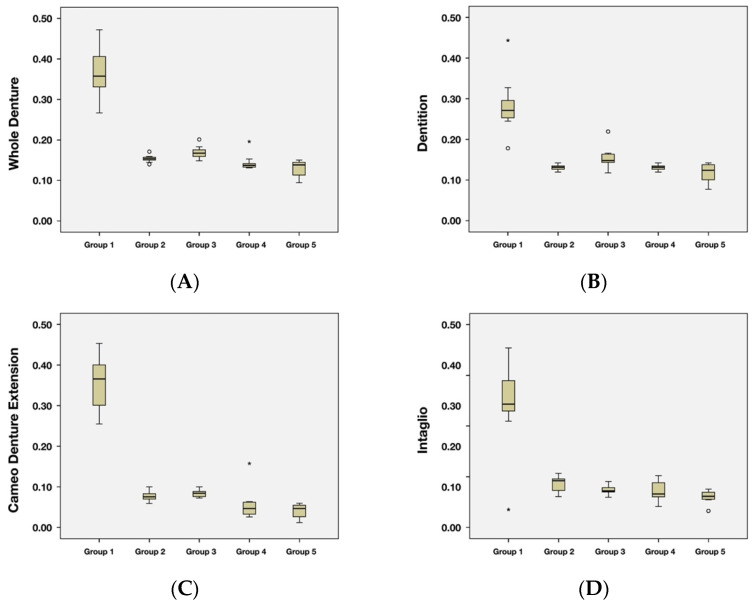
Box plot charts of RMS values. (**A**) Whole denture. (**B**) Dentition. (**C**) Cameo denture extension. (**D**) Intaglio.

The Shapiro–Wilk normality test was used to assess the data normality. All of the *p*-values of datasets were greater than 0.05, and the datasets were deemed as normally distributed. Levene’s Test for Equality of Variances was used to test the assumption of homogeneity of variance. All of the *p*-values of datasets were greater than 0.05, and it was concluded that the assumption of homogeneity of variance was met. Descriptive statistics outcomes were shown in Figure 3 with boxplots.

The multivariate analysis of variance (MANOVA) was used to determine the effect of groups on the whole denture and across the dentition, cameo denture extension, and intaglio portions. It was found that there was an effect of groups (*p* < 0.05). Statistical analyses confirmed that the duplicating protocol had a significant effect on the trueness for all of the tested surfaces and the whole denture. Since there was a significant effect of groups (via MANOVA), the univariate analysis (One-way ANOVA) was used to determine the effect of groups on each intaglio RMS, Flange RMS, Teeth RMS or overall Occlusal. The univariate analysis shows that there was a significant effect of groups on the whole denture and across dentition, cameo denture extension, and intaglio portions (*p* < 0.0001). After the univariate analysis, Tukey’s post-hoc HSD test was used for mean comparisons among the groups. The comparisons were shown in Table 2.

The conventional duplication method (Group 1) was least true with the largest RMS (mean, 95% CI) in the whole denture (0.37, 0.35–0.39 mm) and across dentition (0.28, 0.26–0.30 mm), cameo denture extension (0.45, 0.43–0.47 mm), and intaglio portions (0.34, 0.32–0.37 mm) comparisons. The four digital duplication protocols (Groups 2, 3, 4, and 5) yielded similar trueness values across the dentition, cameo denture extension, and intaglio areas, yet they had a significant difference in the whole denture comparison. Group 3 showed a significantly greater RMS of whole denture (0.17, 0.15–0.19 mm) than Group 5 (0.13, 0.11–0.15 mm).

The labor time spent (mean ± SD, recorded in minutes) for the five groups were summarized in Table 3. The conventional protocol required longer trimming and finishing time (7.55 ± 1.02 min), as well as total labor time (27.64 ± 1.72 min) than the other four digital techniques. Among the four digital groups, the trimming and finishing time (Group 2: 1.48 ± 0.08 min; Group 4: 1.53 ± 0.12 min) and total labor time spent (Group 2: 7.03 ± 0.40 min; Group 4: 7.52 ± 0.57 min) in the DLP 3D-printer groups (MAX X43; Asiga, Alexandria, Australia) were significantly less than those in the SLA 3D-printer groups (Form 2; Formlabs Inc., Somerville, MA, USA). No statistically significant difference was found between the digitization time spent with the CBCT imaging protocol (5.55 ± 0.41 min) and laboratory scanner scanning protocol (5.98 ± 0.47 min).

**Figure 4:**
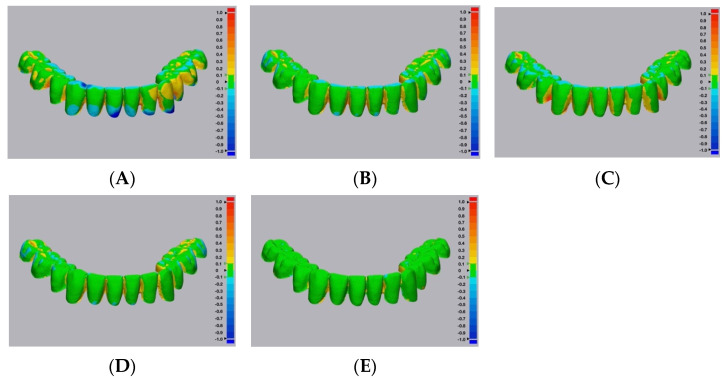
Representative surface matching color maps from the five study groups, across dentition portions. (**A**) Group 1—conventional technique. (**B**) Digital—CBCT—DLP 3D printer. (**C**) Digital—CBCT—SLA 3D printer. (**D**) Digital—Laboratory Scanner—DLP 3D printer. (**E**) Digital—Laboratory Scanner—SLA 3D printer.

**Figure 5:**
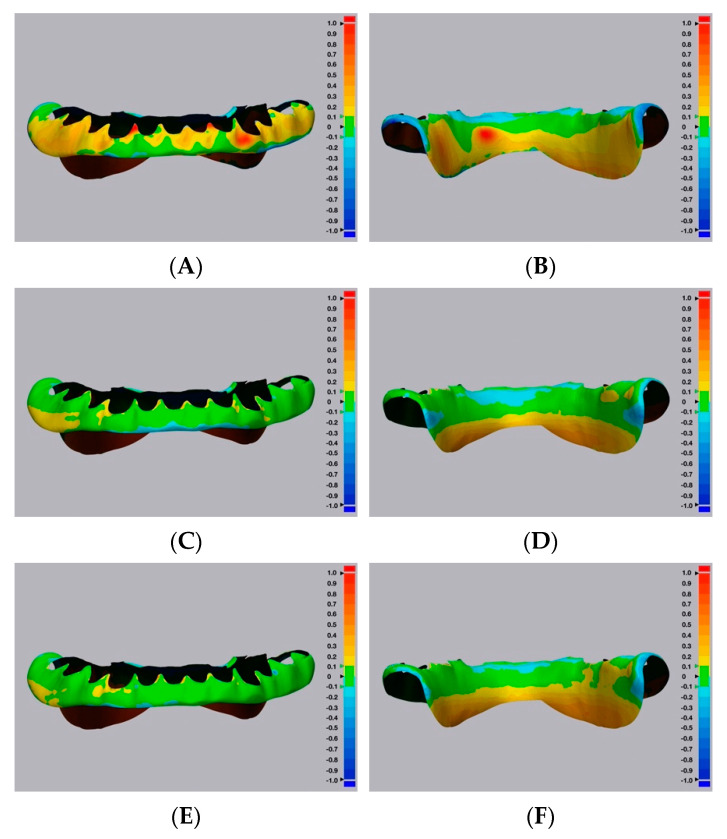

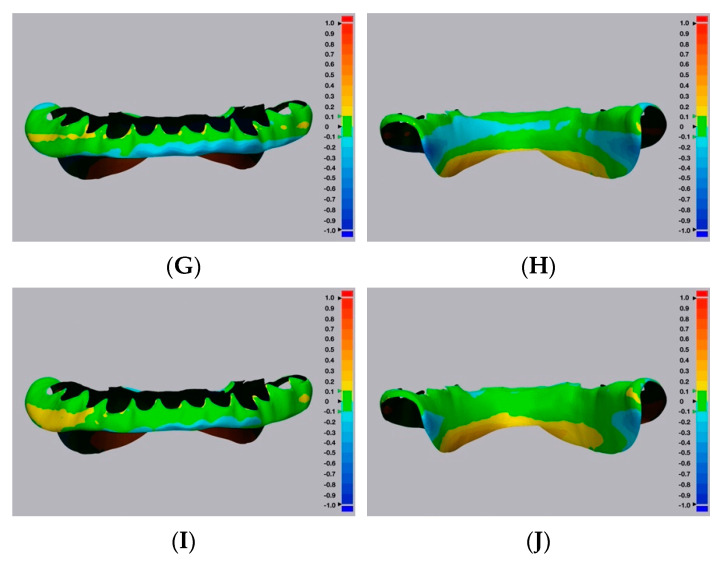
Representative surface matching color maps from the five study groups, across cameo denture extension portion. (**A**,**B**) Group 1—conventional technique. (**C**,**D**) Digital—CBCT—DLP 3D printer. (**E**,**F**) Digital—CBCT—SLA 3D printer. (**G**,**H**) Digital—Laboratory Scanner—DLP 3D printer. (**I**,**J**) Digital—Laboratory Scanner—SLA 3D printer.

**Figure 6:**
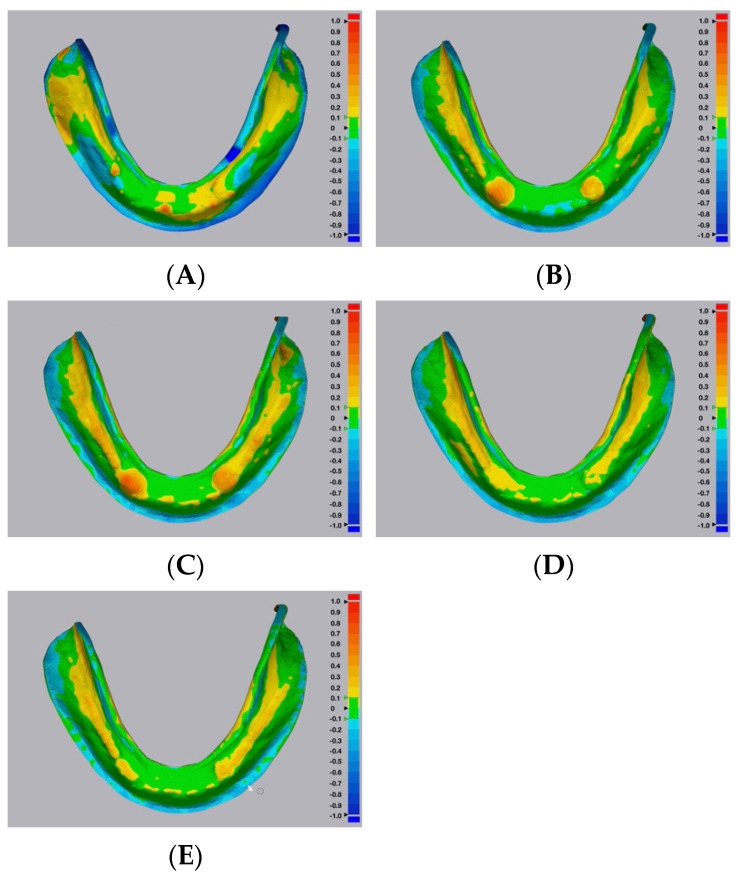
Representative surface matching color maps from the five study groups, across the intaglio portion. (**A**) Group 1—conventional technique. (**B**) Digital—CBCT—DLP 3D printer. (**C**) Digital—CBCT—SLA 3D printer. (**D**) Digital—Laboratory Scanner—DLP 3D printer. (**E**) Digital—Laboratory Scanner—SLA 3D printer.

Color maps of the surface matching differences across dentition, cameo denture extension, and intaglio portions among the five groups are shown in Figure 4, Figure 5 and Figure 6, respectively. Areas in green color are most accurate within ± 0.1 mm in dimensional difference. The positive deviation was represented with yellow to red colors, indicating that the study samples were larger than the corresponding reference files. The negative deviation was represented with a blue color, indicating that the study samples were smaller than the corresponding reference files [30]. At the dentition comparison (Figure 4), Group 1 (Conventional group) showed the most negative deviation (blue color) at the occlusal surface. Groups 2 and 3 (Digital–CBCT groups) also showed some negative deviation at the occlusal surface, while Groups 4 and 5 (Digital-Laboratory Scanner groups) showed the least. At the intaglio comparison (Figure 6), all of the groups showed positive deviations.

## 4. Discussion

The trueness and efficiency of conventional and digital methods for complete denture duplications were compared in this study. The null hypothesis was rejected, confirming that trueness and efficiency to duplicate complete dentures were affected by the duplication techniques (combination of data acquisition techniques and manufacturing techniques and materials). The conventional duplication technique produced duplicated complete dentures, which were less true in all of the tested surfaces (whole denture and across dentition, cameo denture extension, and intaglio surfaces) (Table 2). Furthermore, the conventional duplication technique was less efficient requiring longer trimming and finishing time, as well as total labor time than digital techniques.

The conventional duplication technique was characterized by the manual mold creation with irreversible hydrocolloid impression material and duplication flask, and manual pouring of clear acrylic resin. The trueness and efficiency of using this commonly seen conventional technique are affected by a variety of factors and it is a technique sensitive procedure. Dehydration of impression material and voids in the material mix may distort the duplicated dentures [11]. After the mold was created, the clear acrylic resin should be mixed in an accurate powder/liquid ratio and poured promptly and carefully to the mold. Polymerization shrinkage of clear acrylic resin cannot be avoided during this process. In addition, excessive resins are frequently found around the duplicated dentures, and trimming should be performed with great caution without damaging them. Any mishandling that occurs during these steps would negatively affect the duplication outcomes.

In the present study, trueness comparisons were conducted for the whole denture and across the dentition, cameo denture extension, and intaglio portions separately. The rationale for these analyses is that the trueness for each area could be identified and compared. The trueness outcome from the digital technique groups (Groups 2, 3, 4, and 5) was affected by the combination of data acquisition techniques and manufacturing techniques and materials. Statistical analyses showed that the four digital duplication techniques had similar trueness across the dentition, cameo denture extension, and intaglio portions, yet they had a significant difference in the whole denture comparison. The only significant difference in the whole denture comparison was found between Group 3 (0.17, 0.15–0.19 mm) and Group 5 (0.13, 0.11–0.15 mm), and the difference (about 0.04 mm) could be negligible in the clinical setting. To eliminate the effect of build orientation on 3D-printing accuracy, all of the study samples were 3D-printed with an occlusal plane parallel with the build plate. When the same 3D printer and light-polymerizing resin were used, the laboratory scanner groups (Groups 4 and 5) tended to have smaller RMS values. The additional surface detail of the denture might have been captured by the laboratory scanner (optical scanner), while some surface characteristics may be lost during the CBCT imaging and DICOM-to-STL file conversion process.

Representative color maps showed that, at the dentition comparison (Figure 4), Group 1 had the most negative deviation (blue color) at the occlusal surface. Similarly, Groups 2 and 3 showed some negative deviation at the occlusal surface, while Groups 4 and 5 showed the least. When using the duplicated CDs to record the maxillomandibular relationship and occlusal scheme, negative deviations at the occlusal surfaces may result in the decrease of occlusal vertical dimension. Representative color maps showed that, at the intaglio comparison (Figure 6), all of the groups showed positive deviations. Positive deviations at the intaglio surfaces could indicate gaps between duplicated CD bases and oral tissues, which could affect the clinical stability and retention of the duplicated CDs [30].

The labor time spent is another key factor that clinicians should consider when selecting a denture duplication technique. In this study, the polymerization time of the clear acrylic resin in the conventional technique group (Group 1) and the 3D-printing time in the digital technique groups (Groups 2, 3, 4, and 5) were not considered as manual labor time. The conventional technique required longer trimming and finishing time (7.55 ± 1.02 min), as well as total labor time (27.64 ± 1.72 min) than the other four digital techniques. Additional manual steps were required for the conventional technique and each step required a certain amount of labor time. On the contrary, both CBCT and laboratory scanner scanning were quite efficient, and the time spent in digital files processing was short, as well. Finally, trimming and finishing for digitally duplicated dentures was significantly easier. Particularly, in the DLP 3D-printer groups (Groups 2 and 4), 0.3 mm touchpoint sizes were used and the trimming and finishing time (Group 2: 1.48 ± 0.08 min and Group 4: 1.53 ± 0.12 min) were significantly shorter than the other groups. In the conventional technique group (Group 1), a large amount of excessive acrylic resin had to be removed, and the trimming and finishing time (7.55 ± 1.02 min) was significantly longer.

The limitations of this study have to be taken into account. Only one conventional technique was evaluated, but the trueness outcomes could vary among the different conventional techniques [21]. In addition, only one optical scan protocol was used to digitize the denture, and thus different optical scanners (such as intraoral scanners) can be considered in future studies [31]. A CD can be fabricated by subtractive (milling) or additive (3D printing) manufacturing technologies, and only two additive manufacturing technologies were evaluated in the study. The mandibular master reference CD used in this study had minimal undercuts and was easy to be scanned with an optical scanner (laboratory scanner). For the complete dentures with significant undercuts and long denture extensions, the optical scanning may not be possible. Various digitization and manufacturing protocols can be evaluated in conjunction with complete dentures with different morphologies. Precision values and other comparison softwares can be included in future studies [31].

There are numerous clinical advantages of fabricating duplicated complete dentures, such as reduced overall treatment time for patients, increased patient acceptance (geriatric patients who have limited ability to adapt to a new prosthesis), enhanced neuromuscular adaptation to new dentures, and maintenance of tooth arrangement and occlusal vertical dimension [32]. Digital duplication protocols produced duplicated complete dentures with better trueness and shorter manual labor time in the process. Based on the findings of this study, clinicians can identify the patients who may have difficulty adapting to a new prosthesis, are unwilling to wear new complete dentures or have limited ability and availability to the dental offices. In these cases, the digital duplication protocols discussed in this study can provide valuable ways to produce duplicated complete dentures with high trueness efficiently.

## 5. Conclusions

Within the limitations of this study, the following conclusions can be drawn:

1. Complete dentures duplicated by the conventional duplication method with denture duplicating flask, irreversible hydrocolloid impression material, and autopolymerizing acrylic resin were less true than those fabricated by digital techniques in the whole denture and across the dentition, cameo denture extension, and intaglio portions.
2. Digitization of complete dentures can be achieved by a laboratory scanner or a CBCT scanner. Four digital duplication protocols yielded similar trueness values across the dentition, cameo denture extension, and intaglio areas, yet they had a significant difference in the whole denture comparison. However, the difference could be negligible in the clinical setting.
3. The conventional duplication technique was less efficient requiring longer trimming and finishing time, as well as total labor time than digital techniques. The trimming and finishing time and total labor time spent in the DLP 3D-printer groups were significantly less than those in the SLA 3D-printer groups.

## Figures and Tables

**Table 1:** Characteristics of the research groups.

Group	Sample Size	Data Acquisition Techniques	Manufacturing Techniques and Materials
1	10	Conventional–Alginate ^1^ and Duplication Flask ^2^	Manual and Clear acrylic resin ^3^
2	10	Digital–CBCT ^4^	DLP 3D-Printer ^5^ and Light-polymerizing resin ^6^
3	10	Digital–CBCT ^4^	SLA 3D-Printer ^7^ and Light-polymerizing resin ^8^
4	10	Digital-Laboratory Scanner ^9^	DLP 3D-Printer ^5^ and Light-polymerizing resin ^6^
5	10	Digital-Laboratory Scanner ^9^	SLA 3D-Printer ^7^ and Light-polymerizing resin ^8^

^1^ Jeltrate regular set; Dentsply Sirona, York, PA, USA. ^2^ Denture Duplicator—Flask; Lang Dental Manufacturing Company Inc., Wheeling, IL, USA. ^3^ Caulk Orthodontic Resin; Dentsply Sirona, York, PA, USA. ^4^ 3D Accuitomo 170; J. Morita USA, Irvine, CA, USA. ^5^ MAX X43; Asiga, Alexandria, Australia. ^6^ VeriGuide OS Resin; Whip Mix, Louisville, KY, USA. ^7^ Form 2; Formlabs Inc., Somerville, MA, USA. ^8^ Surgical Guide Resin; Formlabs Inc., Somerville, MA, USA. ^9^ 7Series; Straumann, Andover, MA, USA.

**Table 2:** Root mean square (RMS, measured in mm) comparisons among groups, mean (95% CI).

Groups	RMS			
	Whole Denture	Dentition	Cameo Denture Extension	Intaglio
1	0.37 (0.35–0.39) ^a^	0.28 (0.26–0.30) ^a^	0.45 (0.43–0.47) ^a^	0.34 (0.32–0.37) ^a^
2	0.15 (0.14–0.17) ^b,c^	0.13 (0.11–0.15) ^b^	0.18 (0.15–0.20) ^b^	0.19 (0.16–0.21) ^b^
3	0.17 (0.15–0.19) ^b^	0.15 (0.13–0.18) ^b^	0.19 (0.16–0.20) ^b^	0.18 (0.15–0.20) ^b^
4	0.14 (0.13–0.16) ^b,c^	0.13 (0.11–0.15) ^b^	0.16 (0.13–0.18) ^b^	0.17 (0.15–0.20) ^b^
5	0.13 (0.11–0.15) ^c^	0.12 (0.10–0.14) ^b^	0.14 (0.12–0.16) ^b^	0.16 (0.13–0.19) ^b^

Values indicated by the same superscript letter in each column were not significantly different at α = 0.05.

**Table 3:** Labor time spent (mean ± SD, recorded in minutes).

Group	Mold Creation	Resin Mixing, Pouring, and Cleaning	Digitization	Trim and Finish	Total Time
1	15.23 ± 0.86	4.86 ± 0.49	—	7.55 ± 1.02 ^a^	27.64 ± 1.72 ^a^
2	—	—	5.55 ± 0.41 ^a^	1.48 ± 0.08 ^b^	7.03 ± 0.40 ^b^
3	—	—	5.55 ± 0.41 ^a^	4.82 ± 0.40 ^c^	10.34 ± 0.76 ^c^
4	—	—	5.98 ± 0.47 ^a^	1.53 ± 0.12 ^b^	7.52 ± 0.57 ^b^
5	—	—	5.98 ± 0.47 ^a^	4.79 ± 0.56 ^c^	10.78 ± 0.82 ^c^

SD: Standard deviation; Resin mixing includes mixing and pouring the resin, closing the flask, and removing the excess of resin; Labor time denotes that the same superscript letter (a, b, c) in the same column is not statistically different at α = 0.05.

## Data Availability

The data presented in this study are available on request from the corresponding author. The data are not publicly available due to subsequent ongoing research projects.
